# Identification and validation of anoikis-associated gene SNCG as a prognostic biomarker in gastric cancer

**DOI:** 10.18632/aging.204626

**Published:** 2023-03-30

**Authors:** Yi Li, Qin Pan, Mingxia Cheng, Zhengyuan Wu

**Affiliations:** 1 Department of Operating Room, The First People’s Hospital of Linping District, Hangzhou, Zhejiang 311199, China; 2 Department of Anesthesiology, The First People’s Hospital of Linping District, Hangzhou, Zhejiang 311199, China; 3 Department of Hand Plastic Surgery, The First People’s Hospital of Linping District, Hangzhou, Zhejiang 311199, China

**Keywords:** anoikis, gastric cancer, SNCG, risk signature, immune cell infiltration

## Abstract

As a type of cell apoptosis, anoikis is caused by cells detachment from the extracellular matrix and anoikis resistance is central to cancer metastasis. Here, SNCG was identified as hub anoikis-associated gene in GC and associated with prognosis of patients with GC. To screen the hub anoikis-associated genes connected to GC, the database of Cancer Genome Atlas (TCGA) was employed. For further validating these identified genes, the Gene Expression Omnibus (GEO) dataset was applied, and Western blotting and quantitative Real-Time PCR were carried out. To Identify hub genes, we conducted the analyses of univariate Cox regression, differential expression, and weighted gene co-expression network analysis (WGCNA). According to the identified hub genes, we constructed a model of prognosis. Following complex analysis, SNCG was finally identified as hub anoikis-associated gene in GC. Indeed, K-M and receiver operating characteristic analyses suggested that the expression patterns of SNCG can be used as prognostic factors for GC survival. The expression and survival trends of SNCG were verified in the validation cohort and *in vitro* experimental analyses. The analysis of immune cell infiltration showed that the infiltrated immune cells varied among patients with GC and gene SNCG. Furthermore, due to the significant association of the constructed risk signature with patient age and survival, this risk signature can be used to predict the prognosis of GC. We suggest that SNCG was served as hub anoikis-associated gene in GC. Meanwhile, SNCG may have prognostic potential for overall patient survival.

## INTRODUCTION

As the third leading cause of death associated with cancers, gastric cancer (GC) has fifth largest incidence rate worldwide and is considered as a serious threat to human health [1, 2]. Many epigenetic and genetic factors, as well as multi-step processes, participate in the occurrence and development of GC [3]. In GC patients, the infection of *Helicobacter pylori* is frequently observed [4]. Most of the GC patients are identified at the advanced stage because of no specific symptoms is available for GC diagnosis, thus resulting in a poor 5-year survival rate [5]. Currently, surgical resection is also commonly used as an optimal treatment for GC patients, but only 20%–25% 5-year survival rate is obtained. Additionally, local and systemic recurrence was observed in half of the GC patients who were subjected to adjuvant therapy [6]. To identify the biomarkers for the prediction of prognosis and personalized therapy for GC patients, many studies were conducted. While these studies only identified a few biomarkers with clinical significance. Thus, screening, identifying, and validating new biomarkers for the accurate prediction of GC patients’ prognosis is urgently needed.

Apoptosis is a central part of organismal protection mechanisms; it can prevent the abnormal proliferation of detached cells by inhibiting re-adherence. As another type of cell apoptosis, anoikis was originally discovered in endothelial and epithelial cells. It is caused by cells detached from the extracellular matrix (ECM) and is believed to play a vital role in tissue homeostasis and development. Recently, studies have reported that anoikis also participates in the detachment of various cancer cells, including endometrial carcinoma [7], lung cancer [8], breast carcinoma [9], and also gastric carcinoma [10], from the ECM during metastasis. This indicates that anoikis significantly participates in the development, progression, and distal metastases of cancer, and has the potential to be a hallmark of cancer. For example, the anoikis-associated genes KLF5 and FAIM2 are associated with the prognosis of colorectal and lung cancer, respectively, and silencing KLF5 and FAIM2 can significantly inhibit cancer cell anoikis resistance and proliferation [11]. L1CAM, another anoikis-associated gene, can also promote anoikis resistance and influence the prognosis of endometrial carcinoma patients by boosting epithelial-mesenchymal transformation [7].

Although anoikis is demonstrably relevant to tumor prognosis and progression, its specific value in GC has not been closely analyzed. In this study, to identify the hub genes associated with anoikis in GC patients and enhance the discriminatory ability of highly connected genes, the analyses of univariate Cox regression, differential expression, and GCNA were conducted. Based on the hub anoikis-associated genes, immune infiltration, functional enrichment, and ceRNA regulatory network analyses were performed in GC, and a risk signature was constructed to explore the value of anoikis-associated genes in prognostic prediction, aiming to provide a novel predictive prognostic tool for patients with GC.

## RESULTS

### Identification of hub AG in GC

**Figure 1:**
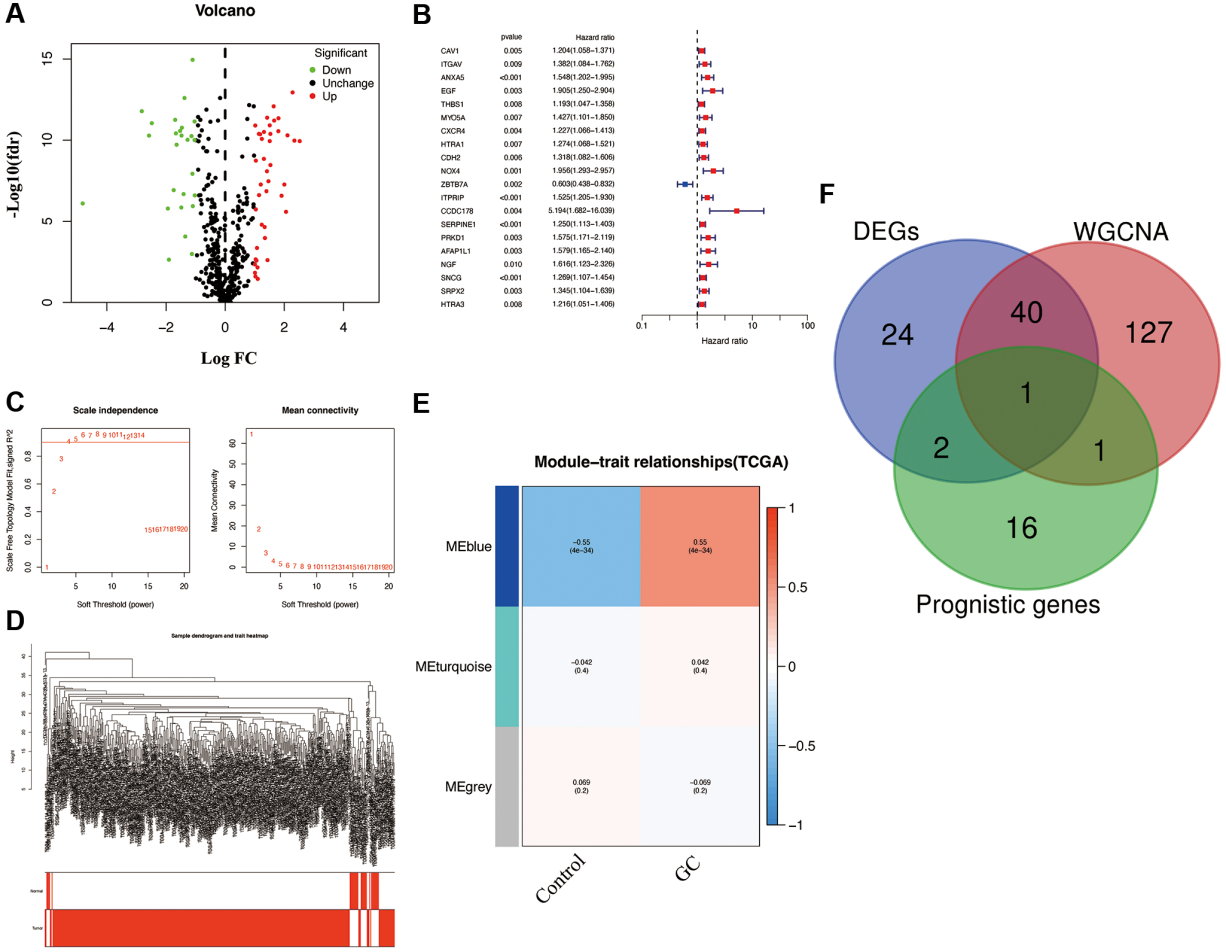
**Characterization of candidate AGs in GC.** (**A**) Volcano plot of differentially expressed AGs with FDR < 0.05 and |logFC| > 1. (**B**) Univariate Cox regression analysis showed prognostic AGs with *p* < 0.01. (**C**) Soft-thresholding powers scale-free fit index. (**D**) Clustering dendrogram of GC samples. (**E**) Heatmap showing the correlation between clinical traits and gene module. Each module was assigned with different colors. The correlation coefficient decreased in size from red to blue. (**F**) The Venn diagram of genes among DEGs, prognostic genes, and WGCNA lists.

By setting the cutoff value at |log FC| > 1 and *p* < 0.05, 67 differentially expressed AGs (DEGs; including 26 downregulated and 41 upregulated AGs) in GC samples were selected from the differential expression analysis (Figure 1A). To identify the AGs associated with prognosis, the analysis of univariate Cox regression as carried out. and Through screening, we identified 20 AGs significantly associated with prognosis (Figure 1B). To further evaluate the functional clusters associated with patients with GC, WGCNA was also performed on the expression profile of 501 downloaded AGs. Based on the scale-free R^2^ = 0.90, the best soft-threshold power was determined as β = 4 (Figure 1C), and three modules, including blue, grey, and turquoise, were identified in WGCNA (Figure 1D). The closest connection between the two clusters was presented as the blue module (r = −0.55, *p* < 0.05), therefore, we selected the AGs in the blue module for further analysis (Figure 1E). Based on the above analyses, overlapping gene SNCG were ultimately identified as hub AG and used to develop a risk signature in patients with GC (Figure 1F).

### Prognostic and clinical value analyses of SNCG in patients with GC

**Figure 2:**
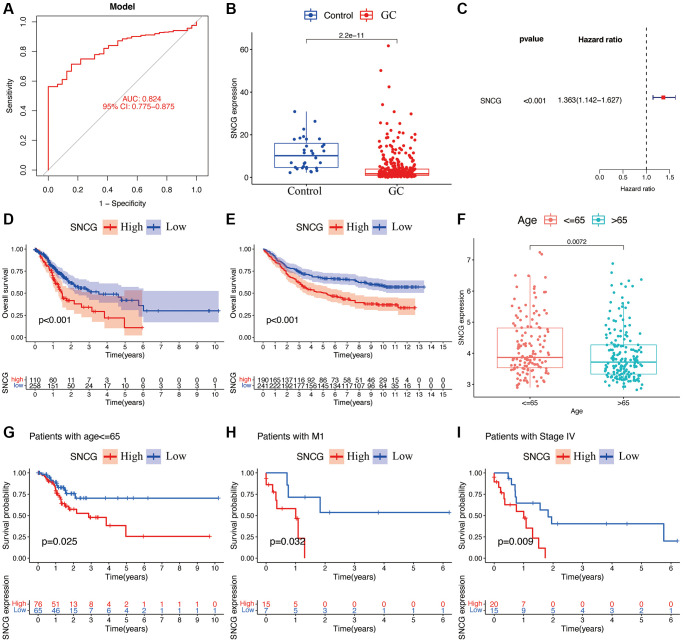
**Functional analyses of SNCG.** (**A**) ROC curves of SNCG. (**B**) Gene expression levels of SNCG in GC samples and normal control. (**C**) Univariate Cox regression of SNCG. Survival curve of SNCG in TCGA (**D**) and GSE84437 (**E**) cohorts. (**F**) Correlations between the expression of SNCG and age. The prognosis of SNCG under the stratifications of age {less than or equal to} 65 (**G**), M1 subtype (**H**), and AJCC stage IV (**I**).

**Figure 3:**
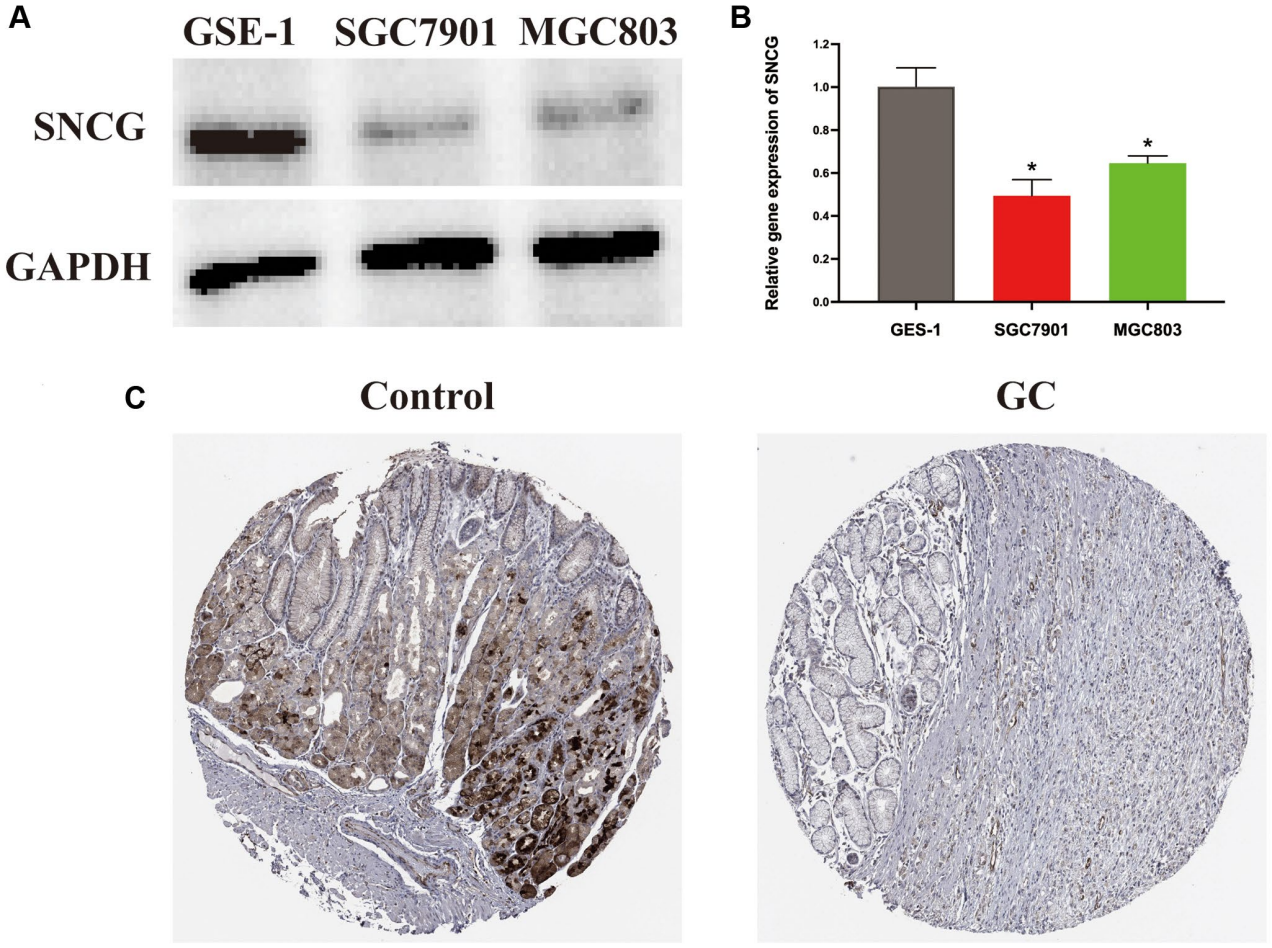
**Expression level of SNCG in GC.** (**A**) Western blot analysis of SNCG. (**B**) QRT-PCR analysis of SNCG. (**C**) Immunohistochemistry data of SNCG from HPA database.

As a single diagnostic biomarker, the area under the ROC curve (AUC) of SNCG was 0.824, indicating that SNCG had a high predictive accuracy in patients with GC (Figure 2A). When evaluating gene expression level of SNCG in GC, we observed that compared to the tissues from health individuals, the tissues from GC patients exhibited significantly reduced SNCG expression (*p* < 0.05; Figure 2B). This result was also confirmed at the translational and transcriptional level by western blot (Figure 3A) and qRT-PCR (Figure 3B) analyses, respectively. For external validation, the results of immunohistochemistry obtained from the Human Protein Atlas (HPA) database were also employed (Figure 3C). To further explore the relationship of SNCG expression with the prognosis of GC, the analyses of KM survival and univariate Cox regression were carried out. The results indicated that SNCG was significantly connected to the overall survival (OS) of GC patients (*p* < 0.05; Figure 2C), meanwhile, the higher expression subgroups of SNCG had a significantly poorer OS (*p* < 0.001) in both TCGA (Figure 2D) and GSE84437 (Figure 2E) validation cohort. Interestingly, GC patients with age >65 had significantly lower SNCG expression level (Figure 2F). Furthermore, we evaluated the value for the prognosis of GC patients with diverse clinical features in clinics. Significant differences between the SNGC high- or low-expressed patients with age ≤65 years (Figure 2G), M1 stage (Figure 2H), and AJCC stage IV (Figure 2I) were observed. In these two subgroups, all high SNCG-expressed subgroup exhibited dramatically poor OS in comparison to those all low SNGC-expressed subgroup.

### Functional enrichment analysis

**Figure 4:**
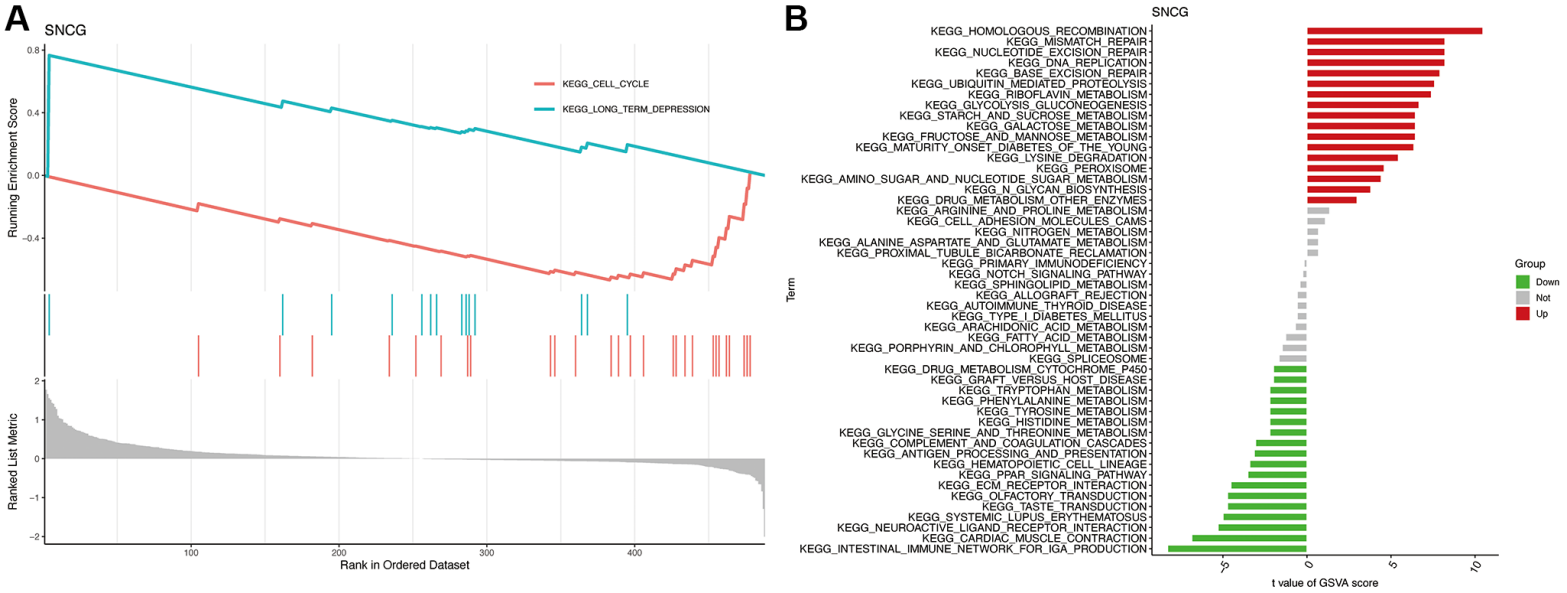
**Functional enrichment analysis of SNCG.** (**A**) GSEA analysis of SNCG. (**B**) GSVA analysis of SNCG.

To explore the potential role of SNGC, we carried out the analyses of GSEA and GSVA. GSEA analysis indicated that the SNCG with the highest enrichment score were significantly connected with pathways related to cell cycle and long-term depression (Figure 4A). GSVA analysis also confirmed that the high SNGC-expressed subgroups were significantly enriched in various cancer- and immune-associated pathways, such as mismatch repair, DNA replication, and intestinal immune network for IgA production pathways (Figure 4B), indicating that the activation of SNCG might participate in modulating cancer and immune progression.

### Immune infiltration analysis

**Figure 5:**
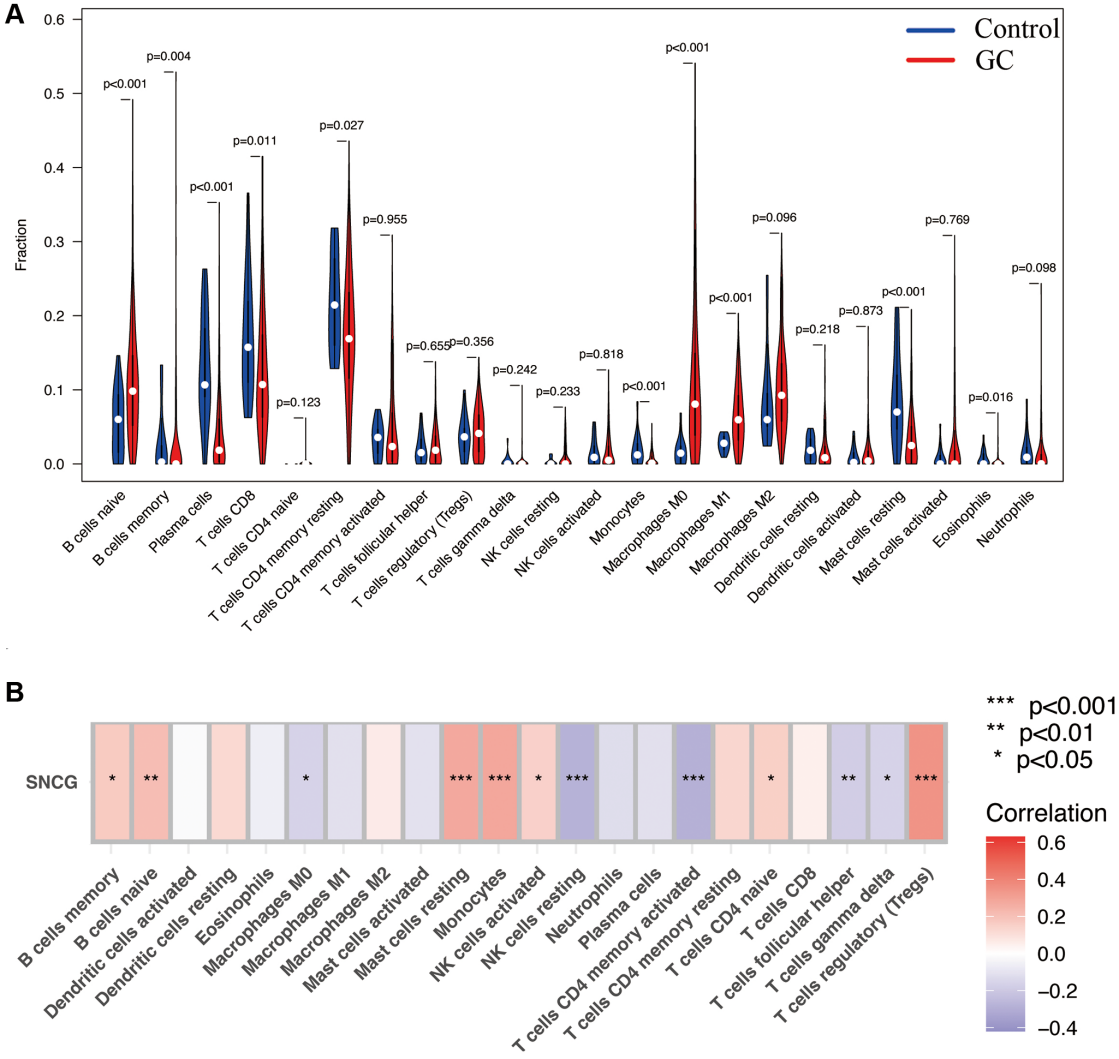
**Immune infiltration analysis.** (**A**) The proportion of 22 types of immune cells between normal control and GC samples. (**B**) Correlation heatmap depicting correlations between infiltrated immune cells and SNCG in GC.

To evaluate the proportion of infiltrated immune cell in GC with a threshold of *p* < 0.05, we applied the CIBERSORT algorithm. As shown in Figure 5A, significantly differential infiltration of several immune cells was discovered between the normal control and GC samples. The results indicated that naïve B cells, M0 macrophages, and M1 macrophages were significantly upregulated in GC samples; however, the proportions of eosinophils, resting mast cells, monocytes, CD4 resting memory T cells, CD8 T cells, plasma cells, and memory B cell, were significantly increased in control samples. Finally, the association of the SNGC expression with infiltration of immune cells in GC was determined. Figure 5B shows that SNCG were strongly associated with the content of immune cells, including Tregs regulator T cells, gamma delta T cells, helper follicular T cells, naïve CD4 T cells, activated memory CD4 T cells, resting NK cells, activated NK cells, monocytes, resting mast cells, M0 macrophages, naïve B cells, and memory B cells, indicating that SNCG may be prognostic targets for GC immunotherapy.

### ceRNA interaction networks

**Figure 6:**
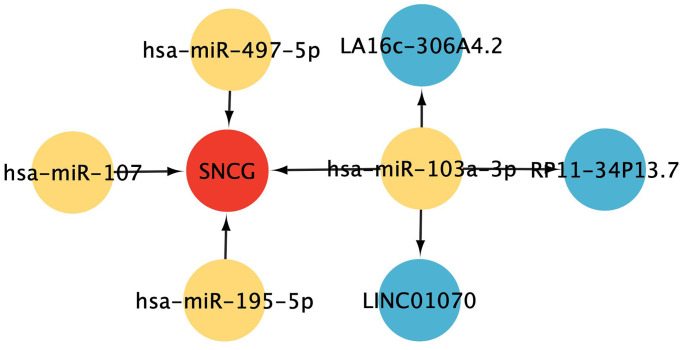
**ceRNA interaction network construction.** Red represents mRNAs, yellow represents miRNAs, and blue represents lncRNAs.

To determine the potential underlying mechanisms of SNCG in GC, a ceRNA network centered on SNCG was generated. The results show that 4 pairs of miRNA-mRNA and 3 pairs of lncRNA-miRNA were included and visualized in Figure 6. SNCG was potentially regulated by miRNAs hsa-miR-497-5p, hsa-miR-107, hsa-miR-195-5p, and hsa-miR-103a-3p, while miRNA hsa-miR-103a-3p might be targeted by lncRNAs LA16c-306A4.2, LINC01070, and RP11-34P13.7.

### Risk signature construction and clinical characteristic analysis

**Figure 7:**
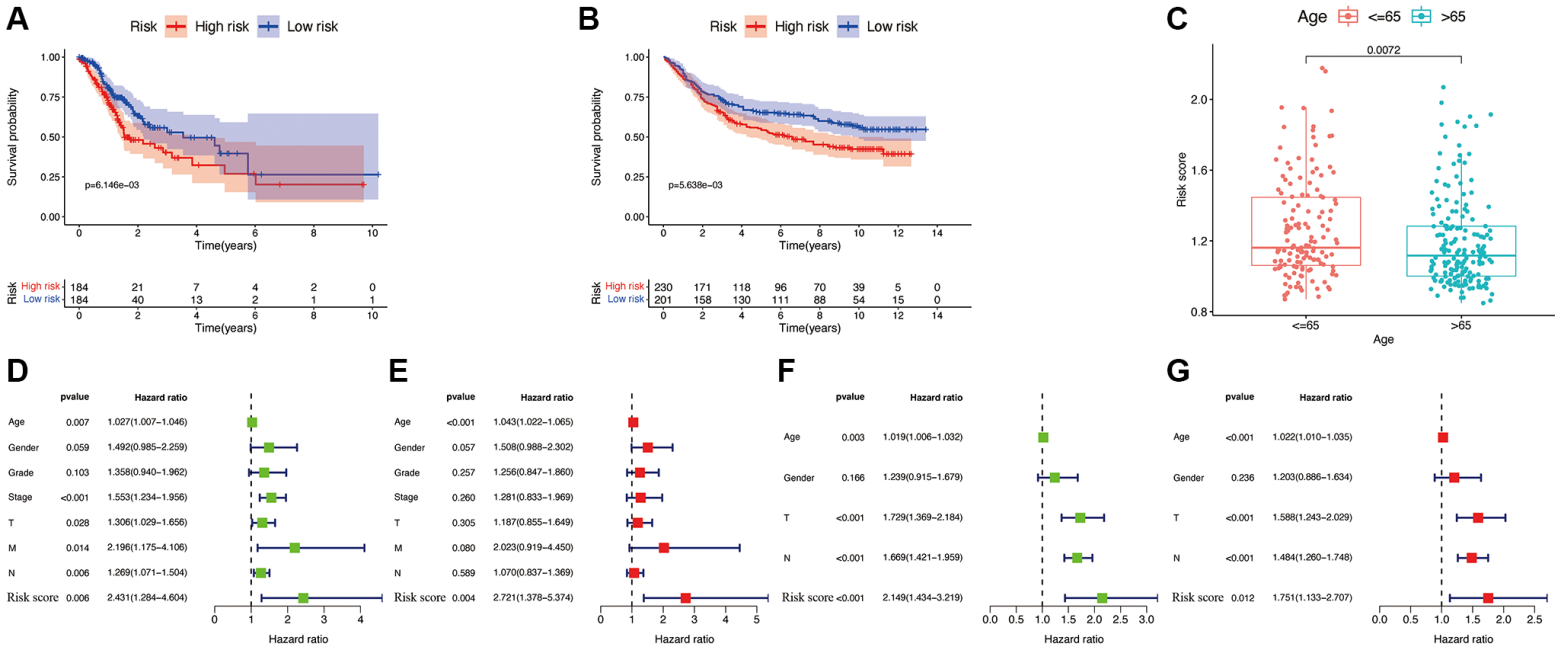
**Efficacy of the constructed model.** Survival curve of the TCGA cohort (**A**) and GSE84437 cohorts (**B**). (**C**) Correlations between the risk score and age. Univariate (**D**) and multivariate (**E**) Cox regressions of clinicopathological features in TCGA cohort. Univariate (**F**) and multivariate (**G**) Cox regressions of clinicopathological features in GSE84437 cohort.

According to SNGC expression, a risk signature was generated in both TCGA and validation cohorts. Meanwhile, according to the median of calculated risk scores, we divided the GC patients into high- and low-risk score subgroups. The patients of the high-risk subgroup significantly associated with worse clinical outcomes in both the TCGA (Figure 7A) and validation (Figure 7B) cohorts (*p* < 0.05). Meanwhile, multivariate and univariate Cox regressions in the TCGA (Figure 7D and 7E) and validation (Figure 7F and 7G) cohorts showed a significant connection of the risk signature to the prognosis of patient, thus serving as an independent prognostic factor in GC. These indicate that the constructed risk signature is significantly related to the prognosis of GC. Additionally, significant association between patients aged ≤65 years with higher risk scores was also found (*p* < 0.05, Figure 7C).

**Figure 8:**
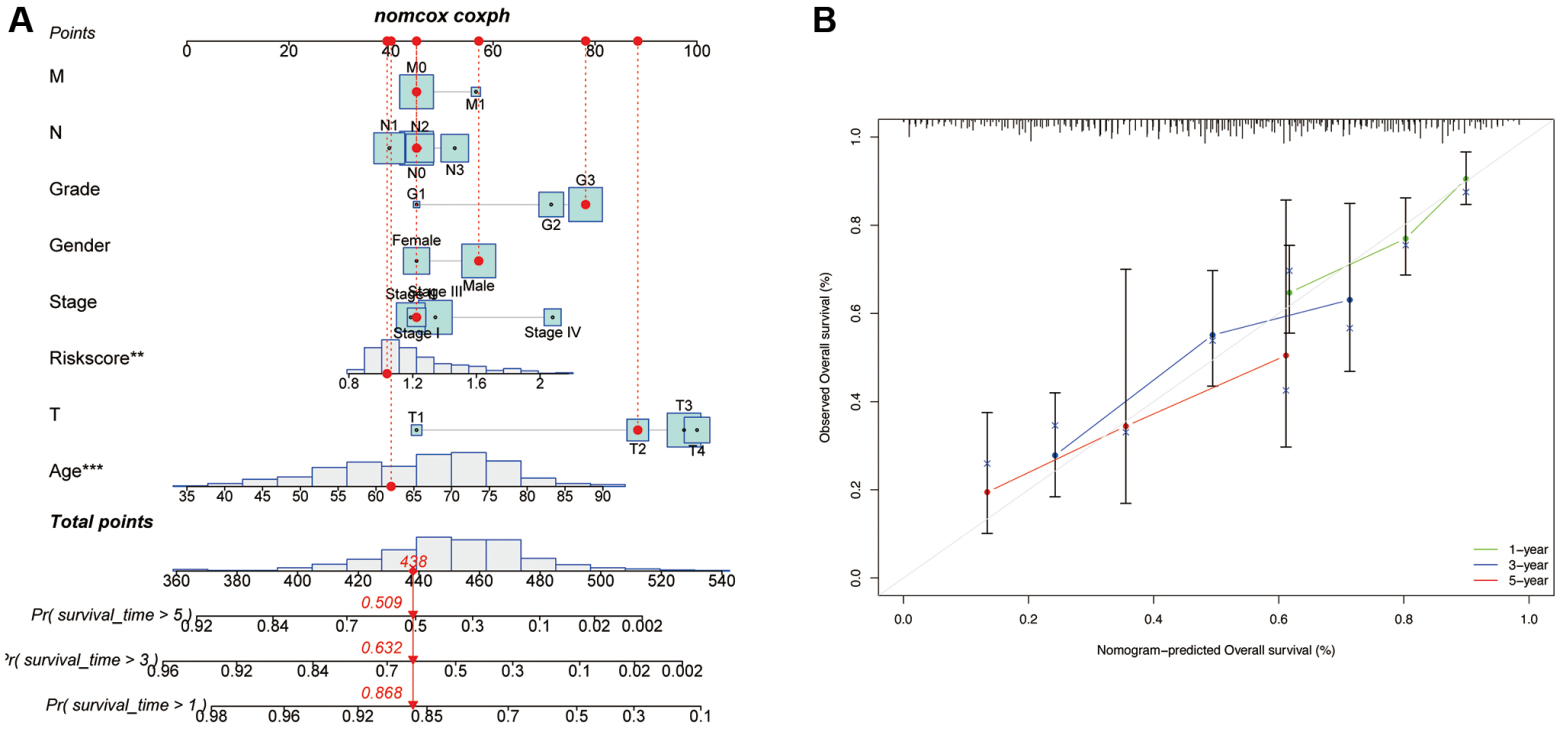
**Development of a nomogram based on risk scores and clinical characteristics.** (**A**) Nomograms to predict the overall survival of GC patients at 1-, 3-, and 5-year using data from TCGA. The red dashed line represented a sample of GC patient’s death probability by year 1, 3, and 5. (**B**) Calibration plot of the nomogram.

Finally, a nomogram plot was established by combining the risk signature and clinical features to exploit the prognostic value of the risk signature (Figure 8A). The results suggested that our established nomogram had a substantial agreement (Figure 8B), which confirmed that our constructed nomogram could potentially be applied for GC prognosis forecasting.

## DISCUSSION

GC is considered as a malignant tumor of digestive system and the heterogeneous characteristics are commonly observed [12]. Recent years, many skills for surgery and techniques for diagnosis are developed, but it’s not sufficient for the diagnosis and treatment of GC in clinics. Therefore, identifying new indicators for the prediction of prognosis and enhancing the accuracy of this prediction for GC are urgently needed. As another type of cell apoptosis, AGs reportedly modulate the biological behaviors of various tumors, such as cell viability and metastasis [13, 14]. AGs can be used as the targets for prognosis and the markers for prospective treatment in cancers.

Herein, using TCGA database, SNGC was identified as a specific AG in GC patients. After that, we generated a robust SNCG-associated risk signature for GC. Then, based on the risk scores, we classified the enrolled patients into two subgroups with high- or low-risk scores. There was a significant difference in prognosis between these subgroups, and using the validation cohort GSE84437, we validated the accuracy of this constructed risk signature. Additionally, the results also indicated that our signature was dramatically correlated with the age of GC samples. To expand the performance of the risk signature, a nomogram combining risk score, age, gender, grades, and TNM stage was generated for patients with GC. Calibration plots showed that our constructed nomogram had a good fit for predicting overall survival.

The identified hub AG SNCG was significantly associated with GC prognosis. Like SNCA and SNCB, SNCG, also named γ-synuclein and breast cancer specific gene 1 (BCSG1), belongs to the family of synucleins [15]. In contrast with SNCA and SNCB, SNCG is not only connected to neurodegenerative disorders, like Alzheimer’s disease and Parkinson’s disease [16], but also primarily relates to various human tumors and participates in cancer occurrence and progression [17]. For example, studies have proved that SNCG is significantly higher expressed in advanced breast cancer samples and peritoneal fluid of endometriosis patients [18], meanwhile, it can facilitate the metastasis of breast cancer [19, 20]. The expression of SNCG is also elevated in high-grade serous patients. Through activating the PI3K/AKT signaling pathway, SNCG can promote ovarian cancer cell metastasis [21]. Furthermore, SNCG can also serve as a potential prognostic gene for biliary tract cancer [22]. While considering the role of SNCG in GC, it has been proved that SNCG relates to the cancer invasion depth and lymph node metastasis of gastric adenocarcinoma [23]. Meanwhile, experiment analyses in GC cells confirmed that SNCG is significantly associated with the growth, apoptosis, and migration of GC [24]. These results indicate that it is of great significance to explore the value of SNCG in GC. In this study, our results discovered that SNCG was significantly related with the prognosis of GC by analyzing TCGA and GSE84437 cohorts. Meanwhile, SNCG was found excellent sensitivity in GC patients from normal control, its sensitivity in GC was 82.4%. This result was similar with the previous study published by Pan et al. [25] that the sensitivity of SNCG in gastric juice and serum from GC patients were 83.91% and 95.40%, respectively. Compared with CA724, a most applied clinical GC biomarker, which only has 65.40% cancer sensitivity [26], our identified gene SNCG showed significant advantages for GC diagnosis. However, this study showed that SNCG was lower expressed in the GC samples than in normal control. This result was inconsistent with previous study that SNCG showed a trend of increased expression in gastric juice and serum of patients with GC [25]. The possible reason is that we detect SNCG at different tissues of GC patients (including gastric juice, serum, and cancer tissue), which may result in inconsistent results on the expression of SNCG. Such difference also exists in other tumors. For example, SNCG was proved connected with the migration of oral squamous cell carcinoma cells [27], whereas it was not significant associated with the metastasis of lymph node [28]. In future exploration, we will expand the sample size and provide more experimental analyses at cellular levels to clarify the specific role of SNCG in GC patients.

Recently, immune treatment has been regarded as a novel therapeutic option for patients with GC, and CIBERSORT analysis is a widely approved method for detecting the relative content of immune cells. In this study, pathway enrichment analyses revealed that SNCG were significantly enriched in immune-associated pathways, such as intestinal immune network for IgA production pathway, and primary immunodeficiency pathway. Additionally, we also confirmed that SNCG was significantly connected with several immune cells, indicating that SNCG might play a virtual role in the immune response in GC and offer a valuable reference for immunotherapy.

Although this study identified hub AG SNCG in GC and proposed a risk signature that displayed a powerful prognostic value in patients with GC, it still had some limitations. First, gene expression and clinical GC cohorts were obtained from TCGA and GEO public websites, and our conclusions should be validated by additional clinical data collected by ourselves. Second, further prospective studies are needed to further confirm the results of our retrospective study. Moreover, the mechanisms underlying the roles of SNGC in the progression of GC should be explored with functional studies in further.

In summary, our study provides insights into the role of hub AG SNCG and develops a novel risk signature for patients with GC. Gene SNCG could improve the accuracy of prognostic prediction and indicate the conditions of immune cell infiltration in GC patients. This study provides a novel perspective for therapeutic improvements in patients with GC.

## MATERIALS AND METHODS

### Data collection and processing

Normal gastric tissues and GC samples RNA sequencing datasets TCGA-GC (GC samples = 375, normal samples = 32) annd GSE84437 (GC samples = 433) with reliable sample sources were obtained from the databases of GEO (https://www.ncbi.nlm.nih.gov/geo/) and TCGA (https://portal.gdc.cancer.gov/). All samples were obtained from Homo sapiens and GSE84437 was selected as the external validation. A total of 501 anoikis-associated genes (AGs) with a relevance score >0.4 were obtained from the GeneCards database. All public databases in this study were searched following the relevant guidelines, and no ethical approval was required from the Ethics Committee of the First People’s Hospital of Linping District.

### Screening for overlapping hub AGs

To improve the highly connected genes discriminatory capacity, univariate Cox regression analysis, WGCNA, and differential expression analysis, were carried out to identify the differentially expressed genes associated with anoikis and were significantly related to GC prognosis. Differential expression analysis for AGs in control and GC samples were conducted using the R package “limma” with the criteria |log fold change (FC)| > 1 and *p* < 0.05. The packages “ggplot2” and “pheatmap” were applied to generate a volcano plot and heatmap, respectively. Meanwhile, to identify the AGs associated with prognosis, the analysis of univariate Cox regression with the cutoff criterion of *p* < 0.01 was conducted. The co-expression network between AGs and sample modules was constructed with the R package “WGCNA”. The best soft threshold was determined to be five as it met the minimum power value with a scale-free topological criterion of >0.90. Thereafter, a topological overlap matrix (TOM) transformed the weighted adjacency matrix, and dendrograms of TOM were constructed using the hierarchical clustering method. To avoid the generation of excessive modules, the major parameters were set as a minModuleSize of 50, deepSplit of two, and height cut-off of 0.25. Finally, AGs with high interconnections were classified into different patterns. The gene significance (GS) and module eigengenes (MEs) were calculated for each module. AGs overlapping between the analyses of WGCN, differential expression, and univariate Cox regression were visualized with package “VennDiagram” and considered as hub AGs for further analysis.

### Clinical analyses of hub AG

The clinical value of hub AG for GC, as well as their specificity and sensitivity, were determined using Kaplan-Meier curves, univariate Cox regression, differential expression, and receiver operating characteristic (ROC) curve methods using various R packages. Based on the identified hub AG expression, GC patients were divided into two subgroups with high- and low-expression. Subsequently, the prognostic value of hub AG for GC patients with different clinical features were tested and visualized by the “survival” package in R. Statistical significance was set at *p* < 0.05. The HPA database was applied to determine the difference of hub AG at protein level.

### Gene set variation analysis (GSVA) and gene set enrichment analysis (GSEA)

GSVA was performed for each gene set and scoring. According to the GSVA score matrix, the changes at the gene-level were converted into changes at the pathway-level by the R package “GSVA”, and the potential biological functions were ultimately evaluated. The C7 and Hallmark gene sets v7.4 were applied in the analysis of GSEA. Enriched gene sets were used to detect KEGG pathways. Gene sets with the adjusted *p*-value less than 0.05 were considered significantly enriched after 1000 substitutions.

### Immune cell infiltration assessment

Using the CIBERSORT algorithm, the proportion of 22 types of immune cell infiltration was determined on TCGA-GC sequencing data. Using the package “vioplot”, we compared the difference of the infiltration of immune cells between GC and normal gastric tissues. Furthermore, the connection of the hub gene expression to the infiltration of immune cells was also quantified based on CIBERSORT analysis, while a correlation heatmap was visualized using the R package “corrplot”.

### ceRNA interactions network construction

Interactions between hub AGs and miRNAs were predicted using miRWalk3.0 (http://mirwalk.umm.uni-heidelberg.de/). Meanwhile, the potential target lncRNAs of identified miRNAs were predicted based on the lncRNASNP3 (http://gong_lab.hzau.edu.cn/lncRNASNP3/#!/). The interactions that were fitted to the TargetScan, miRDB, and miRanda databases were selected to draw the ceRNA networks. Network of ceRNA was constructed and visualized using Cytoscape (version 3.7.1) software.

### Identification of hub AG-associated risk signature

Based on the identified hub AG expression, we calculated the risk score of the hub AG-associated risk signature with the formula:


risk score=∑expgenei×βi

where i and β are regression coefficients, and expgenei is hub AG relative expression.

Based on the calculated risk scores, we separated the GC patients into two subgroups with high- and low-risk scores. To compare the ability of prognosis according to the risk level, the analyses of Cox regression and survival were carried out. For predicting the outcome of GC patients, a nomogram was constructed according to the calculated risk score and GC clinical characteristics, such as age, T stage, AJCC stage, gender, grade, N stage, and M stage, using the package “rms”. To validate our constructed nomogram, the bootstraps with 1000 resamples were employed. The discrimination and accuracy were estimated using calibration curves.

### Cell culture

The GES-1 human gastric mucosal epithelial cells, MGC803 and SGC7901 GC cells were provided by Shanghai Zhong Qiao Xin Zhou Biotechnology Co., Ltd. The FBS (10%)- and penicillin/streptomycin (1%)-contained RPMI-1640 medium (Gibco) was employed to culture the cells at 37°C under 5% CO_2_.

### qRT-PCR

To isolate the RNA from cells, Trizol reagent (Invitrogen) was employed. The RNeasy mini kit (QIAGEN, Valencia, CA, USA) was used to isolate RNA and then RNA was reverse transcribed to cDNA using a Transcriptor First-strand cDNA synthesis kit (Roche). Next, a qRT-PCR was conducted using SYBR green master mix on an Applied Biosystems 7500 Real Time Cycler (Applied Biosystems). The PCR conditions are as follows: 95°C for 10 min, 40 cycles at 95°C for 15 sec and 60°C for 1 min, followed by a standard melting curve. All samples were running in triplicate and gene expression was measured using the 2^−ΔΔCT^ method. GAPDH was used as a normalization control. A list of the primer pairs and their sequences that were used in this study is presented in Supplementary Table 1.

### Western blot analysis

To extract the total protein from cells, phenylmethanesulfonyl fluoride (Beyotime)-contained RIPA Lysis Bufer (Beyotime) was employed. For each sample, 60 μg proteins were separated using 10% SDS-PAGE gel. After transferring of the protein bands to a PVDF membrane, overnight incubation 4°C of the membrane with antibodies against SNGC (Abcam) and GAPDH (Abcam) was performed. Next, after 3-time washing using PBST and 1-hour incubation with DyLightTM800 4X PEG conjugated secondary antibody (Cell Signal Technology), the protein bands were identified by An Odyssey Infrared Imaging System.

### Availability of data and materials

The datasets used and/or analyzed during the current study are available from the corresponding author on reasonable request.

## Supplementary Materials

Supplementary Table 1
