# Supplementary material for: Identification and validation of anoikis-associated gene SNCG as a prognostic biomarker in gastric cancer
Source: Aging (Albany NY). 2023 Mar 30;15(7):2541–53. doi: 10.18632/aging.204626 (PMC10120907; doi:10.18632/aging.204626)
Supplement: Supplementary Table 1 [file aging-15-204626-s001.pdf]

SUPPLEMENTARY TABLE

Supplementary Table 1. Primer sequences used in qRT-PCR experiments.

Gene	Primer	Primer sequence (5′ to 3′)	Product size (bp)
GAPDH	Forward	CTGCCTCGATGGGTGGAGTC	148
	Reverse	GAGTTAAAAGCAGCCCTGGTG	
SNCG	Forward	ACCAAGGAGGGGGTCATGTA	124
	Reverse	GGCCACTATGGGAGGACAC	
